# Sustainable Membrane-Based Wastewater Reclamation Employing CO_2_ to Impede an Ionic Precipitation and Consequent Scale Progression onto the Membrane Surfaces

**DOI:** 10.3390/membranes11090688

**Published:** 2021-09-06

**Authors:** Muhammad Kashif Shahid, Younggyun Choi

**Affiliations:** 1 Research Institute of Environment & Biosystem, Chungnam National University, Daejeon 34134, Korea; mkbutt2000@gmail.com; 2 Department of Environmental & IT Engineering, Chungnam National University, Daejeon 34134, Korea

**Keywords:** CO_2_ utilization, fouling, membrane, pollution, wastewater treatment, circular economy

## Abstract

CO_2_ capture and utilization (CCU) is a promising approach in controlling the global discharge of greenhouse gases (GHG). This study details the experimental investigation of CO_2_ utilization in membrane-based water treatment systems for lowering the potential of ionic precipitation on membrane surface and subsequent scale development. The CO_2_ utilization in feed water reduces the water pH that enables the dissociation of salts in their respective ions, which leave the system as a concentrate. This study compares the efficiency of CO_2_ and other antifouling agents (CA-1, CA-2, and CA-3) for fouling control in four different membrane-based wastewater reclamation operations. These systems include Schemes 1, 2, 3, and 4, which were operated with CA-1, CA-2, CA-3, and CO_2_ as antiscalants, respectively. The flux profile and percent salt rejection achieved in Scheme 4 confirmed the higher efficiency of CO_2_ utilization compared with other antifouling agents. This proficient role of CO_2_ in fouling inhibition is further endorsed by the surface analysis of used membranes. The SEM, EDS, and XRD examination confirmed the higher suitability of CO_2_ utilization in controlling scale deposition compared with other antiscalants. The cost estimation also supported the CO_2_ utilization for environmental friendly and safe operation.

## 1. Introduction

Climate change and the water scarcity are two major challenges of our time [1]. The natural process of climate change has very slow spread over hundreds of centuries. Today, anthropogenic activities are the key contributors in climate change, including the burning of fossil fuels (natural gas, oil, and coal), chemical discharges into the atmosphere, deforestation, and the hasty development in the agricultural and industrial sectors [2,3]. These activities cause excessive discharge of greenhouse gas (GHG) emissions, especially, carbon dioxide (CO_2_), into the atmosphere and disrupts the climate balance [4]. Global warming is one of the major challenges associated with the rising level of GHGs in the atmosphere [5]. Global warming also affects the water cycle by disturbing the water quality and accessibility, by increasing storms and floods, and by amplifying the rate of droughts [6,7].

Several approaches have been introduced to reduce the CO_2_ discharge into the environment such as the utilization of renewable resources (biomass, wind, and solar energy) [8]. However, the two major limitations of power generation from renewable resources are (i) the expensive production process and (ii) the lower availability of renewable resources compared with fossil fuels. CO_2_ capture and storage (CCS) is one of the eminent approaches to balancing the atmospheric CO_2_ level and involves the CO_2_ capture at the source point, compression, and transport to the storage location [9]. CO_2_ capture and utilization (CCU) is another promising method in controlling the global discharge of GHG. Several CCU techniques have been introduced such as cryogenic separation, adsorption, absorption, biofixation, membrane separation, etc. [10,11]. The CCU system is useful not only in controlling GHG effects and global warming but also in generating instinctive financial means. For instance, the wastewater treatment plants (WWTPs) are responsible for generating GHG emissions, specifically CO_2_ during degradation of organic substances [12]; however, CO_2_ can be captured and utilized to inhibit the fouling in membrane operations [13].

Fouling is a phenomenon whereby ions or particles from the feed solution (influent stream) are deposited onto the surface and/or into the interior structure (pores) of membranes in membrane-based water and wastewater treatment systems such as microfiltration (MF), ultrafiltration (UF), membrane bioreactor (MBR), membrane distillation (MD), reverse osmosis (RO), and forward osmosis (FO) [14,15,16]. Fouling results in serious flux drop and compromises the quality of the product water. The excessive membrane fouling may need membrane replacement or intense chemical cleaning, which may increase the operational expenditures [17,18]. In the RO operations, the applied pressure at feed side must be higher than the osmotic pressure of the influent to facilitate the transportation of water molecules from polymeric membrane, whereas other ionic components of influent are rejected [19]. The productivity of the process is restricted by several constraints that unpleasantly influence the membrane efficiency, and regardless of substantial pretreatment procedures for feed water, fouling cannot be completely removed [20].

A recent study reported the utilization of CO_2_ solutions to clean the organic fouling from the RO membranes [21]. This study used the CO_2_ nucleation approach to wash out the organic fouling from the membrane surface. Besides several cleaning approaches, the utilization of antifouling agents and the acidification of feed water are widely adopted approaches for the kinetic control of scale deposition; however, their application is conditioned with toxic byproducts and the threshold levels [22]. We have been introduced to the CO_2_ utilization for scale inhibition in RO processes [23]. Principally, the addition of CO_2_ in feed water causes a significant drop in pH, which facilitates the dissociation of salts in their respective ions and finally discharge in concentrate stream [24]. Earlier, we reported the scale inhibition utilizing CO_2_ in constant flux mode of membrane operation and the major part of the existing literature addresses the same operational schemes [23].

In this study, the process efficiency of membrane-based wastewater treatment systems is examined under constant applied pressure mode. Several antifouling agents were utilized for the inhibition of scale deposition on a membrane surface during operational phase. The major aim of this study is to compare the competency of CO_2_ with other antifouling agents in terms of scale inhibition, flux recovery, salt rejection, and the production of toxic and nontoxic byproducts. This study is beneficial in improving the ecofriendly and sustainable operation of wastewater reclamation plants with CO_2_ utilization.

## 2. Materials and Methods

### 2.1. Membranes and Chemical Agents

The analytical grade chemicals and reagents were obtained from Fisher Scientific and used without further purification. The synthetic wastewater was used as a feed water in membrane-based water treatment systems (Table 1). As the advanced wastewater reclamation plants are the potential beneficiaries of this study, the quality of feed water was adjusted considering the quality of tertiary treated effluent of wastewater treatment plants. The wastewater composition was adjusted by adding an appropriate amount of chemicals in an influent reservoir. The molar concentration of added chemicals included 20 mM of NaCl, 5 mM of CaCl_2_, 3.4 mM of MgCl_2_, 1.14 mM of KCl, 0.30 mM of KNO_3_, 0.08 mM of MgSO_4_, and 0.07 mM of KH_2_PO_4_. The commercial scale antifouling agents (CA-1, CA-2, and CA-3) were acquired from a domestic market in Korea. The basic composition of CA-1 and CA-2 includes the mixture of a varied ratio of polycarboxylic acid and a phosphonic acid derivative. However, CA-3 is composed of polyacrylic acid and 2-phosphonobutane-1,2,4- tricarboxylic acid (PBTC). The spiral wound polyamide thin film composite RO membrane (Hyundai Wacortec, Korea) with a 0.4 m^2^ surface area and a ~100 Da molecular weight cut-off was used in this study. As per manufacturer recommendations, membrane can be operated finely at a maximum of 45 °C operating temperature, 8.6 bar applied pressure, 2 L/min feed flowrate, and 3–10 pH.

### 2.2. Membrane Operations

**Figure 1:**
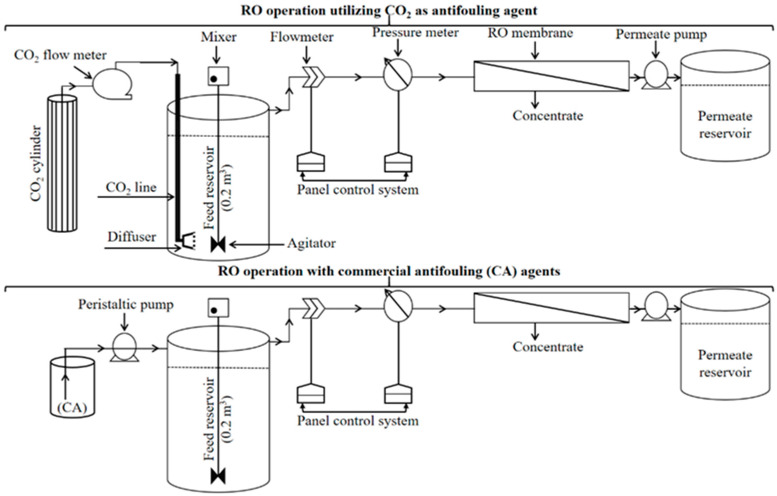
Schematic representation of RO schemes operated with CO_2_ and commercial antifouling (CA) agents.

All of the membranes were rinsed before applying for filtration of the synthetic wastewater. Four single-pass independent RO schemes were designed with different antifouling agents such as CA-1, CA-2, CA-3, and CO_2_ and named Schemes 1, 2, 3, and 4, respectively. All of the schemes were operated at constant applied pressure (4 bar), 40% recovery, 11.32 LMH permeate flux, and 99% salt rejection. The influent pH was 7.25 for Schemes 1, 2, and 3, whereas Scheme 4 was operated after dropping the feed water pH. The feed water of Scheme 4 was conditioned with CO_2_ in a 0.2 m^3^ reservoir with a 300 mL/min injection rate. About a 1.5 min injection time was sufficient to decrease the pH of 0.2 m^3^ feed water from 7.25 to 6. Scheme 4 was operated after stabilizing the pH of the feed water. Figure 1 is a schematic representation of RO systems operated with CO_2_ and commercial antifouling agents.

After certain cycles of filtration, the cleaning in place (CIP) protocol was followed to reinstate the process efficiency in terms of permeate flux and salt rejection capability. The CIP was accomplished utilizing 1% ethylenediaminetetraacetic acid (EDTA), 0.1% sodium hydroxide, and 0.2% HCl solutions. All of the cleaning agents were applied stepwise from the feed side with flowrate of 50 mL/min and 60 min contact time. The operational date comprising permeate flux, salt rejection, pH variation, and the ionic mass balance was concisely monitored during operation. Membrane autopsy was conducted after the termination of all operations of all schemes.

### 2.3. Instrumentation

The pH and conductivity of the water samples were recorded on 96pH-L2 (Samsan Korea Ltd., Yongin, Korea) and EC96 (M-Cubic Co., Ltd., Daejeon, Korea), respectively. The concentration of anions was determined using Thermo Scientific™ Dionex™ ICS-5000, and the cationic concentration was analyzed on Thermo Scientific™ Dionex™ ICS-1000. The characterization of virgin and fouled membranes was conducted by X-ray diffractometer (XRD, Rigaku Corp., Tokyo, Japan) and Scanning Electron Microscopy (SEM, Hitachi SU-70, Hitachi Ltd., Tokyo, Japan) coupled with Energy Dispersive Spectroscopy (EDS).

## 3. Results and Discussion

### 3.1. Operational Performances of RO Schemes

**Figure 2:**
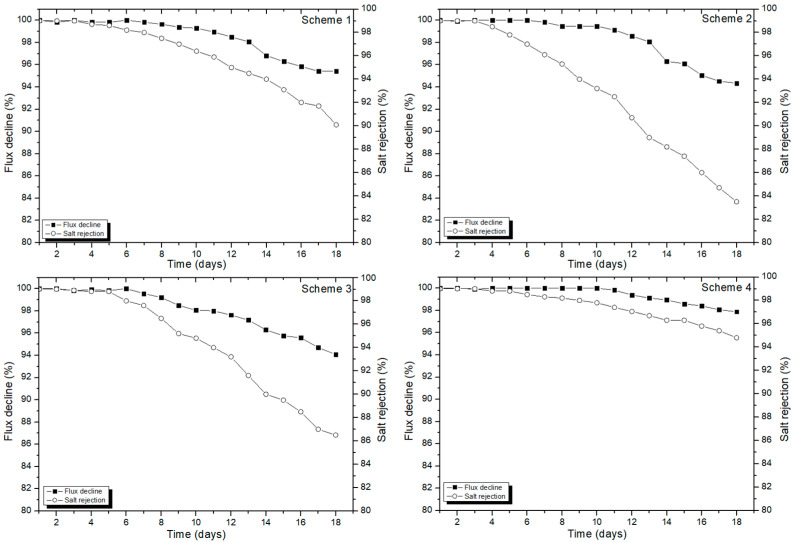
The permeate flux and salt rejection in RO operation for Schemes 1, 2, 3, and 4, operated with CA-1, CA-2, CA-3, and CO_2_ as antiscalants, respectively.

Initially, the operation of all schemes was monitored for 18 days and the system efficiency was determined based on consistency in permeate flux and the percent salt rejection of membranes. Later, the CIP was conducted to estimate the restoration efficiency of all the RO schemes. Figure 2 shows the operational performance of Schemes 1, 2, 3, and 4, which were operated with CA-1, CA-2, CA-3, and CO_2_, respectively. Scheme 1 presented a 5% decline in permeate flux and a 10% reduction in salt rejection. Scheme 2 exhibited 6% loss in initial permeate flux, and the salt rejection dropped from 99% to the 83%, which shows that 16% of salt concentration (in bulk) passed through the membrane during filtration. Similarly, Scheme 3 indicated a 14% loss in salt rejection along with a 6% loss in initial permeate flux. Comparatively, the performance efficiency of Scheme 4 was much better, with less than 2% decline in permeate flux and 94.5% salt rejection.

As the comparative analysis of the operational schemes revealed a prominent difference in performances of all the schemes, the trend for process efficiency of all the systems can be draw as Scheme 4 > Scheme 3 > Scheme 1 > Scheme 2. The operational results confirmed that scale deposition and formation of cake layer on the membrane surface was kinetically controlled in the presence of CO_2_ whereas an inverse performance was expressed by other antifouling agents such as CA-1, CA-2, and CA-3. The higher loss in permeate flux and salt rejection by Scheme 2 is the direct representation of excessive precipitation of inorganic ions on the surface of membrane.

As the membranes are operated in the mode with constant applied pressure, the increasing filtration cycles and, subsequently, ionic deposition onto the membrane surface, influenced the permeability of the RO membranes. The loss in permeate flux and salt rejection appeared due to the continuous deposition on membrane surfaces. It is noteworthy that concentration polarization also contributes to reducing the membrane efficiency after several continuous cycles of filtration. The higher concentration polarization in vicinity of membrane surface caused a higher accumulation of solute concentration at the surface of the membrane, which significantly contributed to the development of the cake layer [25]. As the concentration polarization layer usually forms in conjunction with the membrane surface, the length of feed channels affects the solute concentration. The higher velocity decreases concentration polarization via enhanced mass transfer and reduced yield [26].

Schemes 2 and 3 presented considerable low efficiency in terms of maintaining percent salt rejection by membrane surfaces. This type of finding could be a result of entrenched foulants into the feed spacers of membranes, thereby reducing membrane permeability and subsequent loss in the percent salt rejection [27]. The ionic movement from bulk solute (feed water) to the membrane surface, and the back diffusion of cations and anions settled by cake layer are two major constraints responsible for the loss in permeate flux and percent salt rejection. It can be stated that the high degree of concentration polarization may amplify the osmotic pressure and subsequent loss in the salt rejection. In particular, the solubility limits of divalent ions (e.g., Ca^2+^, Mg^2+^, etc.) can rise, and subsequent ionic precipitation can influence the mass transport phenomena [28].

Beside lowering the pH of feed water, the dissolved CO_2_ molecules also contribute to destruction of fouling layers developed onto the membrane surface. The voids present on the surface of membrane can be assumed as CO_2_ nucleation localities, thus decreasing the free interfacial energy needed for the CO_2_ nucleation. Likewise, the fouling layers developed onto the surface of membrane may also assist as a substrate for CO_2_ nucleation [29]. The dissolved CO_2_ molecules potentially move into the nooks inside the fouling layer triggering nucleation, development, and extrication of CO_2_. Furthermore, an excessive existence of vicinities might be predicted in a porous cake layer compared with thick gel layer, thus assisting CO_2_ nucleation and consequent maintenance of permeate flux.

**Figure 3:**
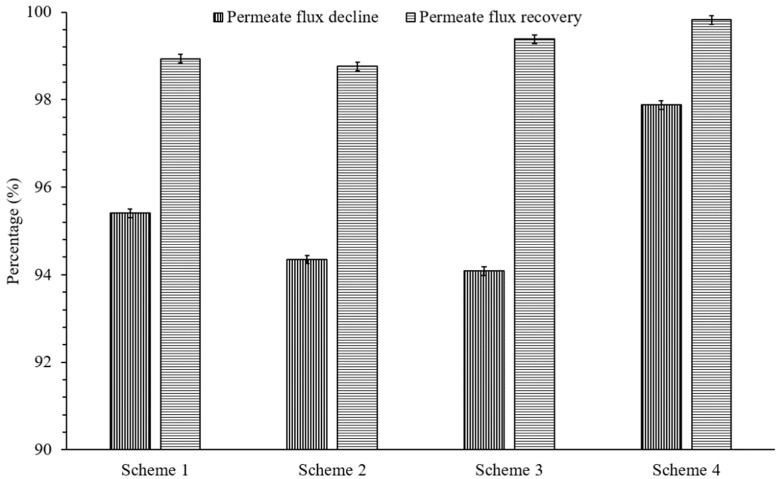
The percentage permeate flux decline and permeate flux recovery after CIP with EDTA (1%), NaOH (0.1%), and HCl (0.2%). Cleaning solutions were introduced from the feed side (50 mL/min flowrate, 25 °C temperature, 1 bar applied pressure, and 1 h contact time for each chemical).

The fouled membranes were cleaned with recommended CIP protocol [13]. The performance of CIP was examined for restoring the permeate flux of membranes that were primarily fouled with influent comprising several monovalent and divalent ions. Figure 3 shows the percentage permeate flux decline for all schemes and, later, the recovery of permeate flux via CIP. Scheme 4 was immensely capable of eliminating deposited foulants from the surface of membrane compared with other schemes. Up to a 2% drop in recovery of the initial permeate flux is estimated for other schemes. The greater recovery of permeate flux in Scheme 4 can be credited to the CO_2_ utilization that proficiently hindered the formation of cake layer or permanent fouling during the filtration process.

### 3.2. Mass Balance Estimation for Monobalent and Divalent Ions

The ionic composition of feed wastewater, product water, and the rejection stream were continuously monitored and utilized for the estimation of ionic mass balance. Equation (1) was utilized to determine the accumulated share of cationic and anionic concentration inside the RO schemes.

ΔM_Acc_ = Q_I_ × C_I_ − Q_P_ × C_P_ − Q_C_ × C_C_ (1)

where ΔM_Acc_ represents the concentration of particular ion accumulated inside the RO system. Q_I_, Q_P_, and Q_C_ represent the influent, permeate, and concentrate flowrate, respectively, whereas C_I_, C_P_, and C_C_ represent the ionic concentration in an influent, permeate, and concentrate stream, respectively.

**Figure 4:**
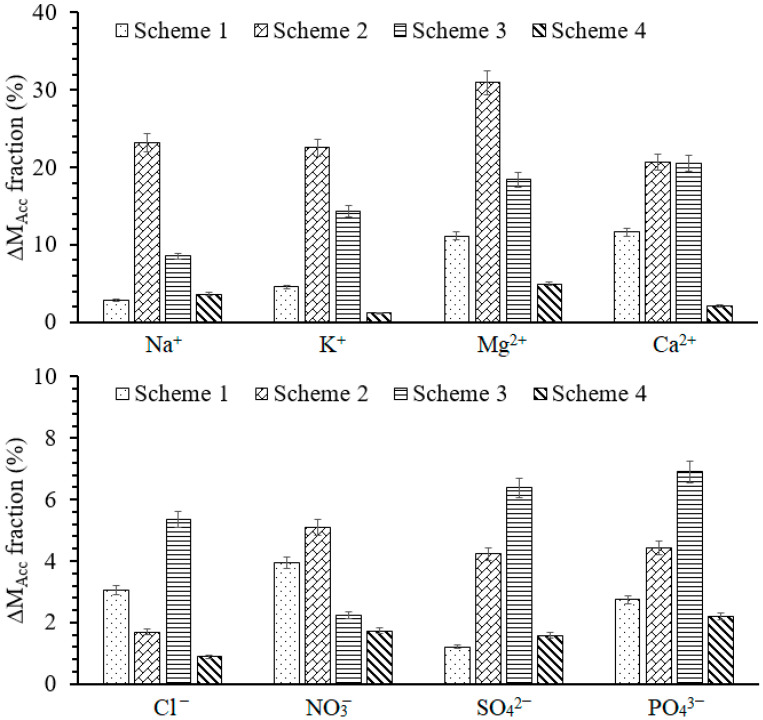
The accumulated mass fraction of cations and anions. The error bars represent the standard deviation of last three sampled days of all operational schemes.

The mass balances of cations (e.g., K^+^, Na^+^, Mg^2+^, and Ca^2+^) and anions (e.g., Cl^−^, NO_3_^−^, PO_4_^3−^, and SO_4_^2−^) were calculated, and the estimated accumulated share of cations and anions is depicted in Figure 4. The accumulated ionic concentration represents the ionic concentration of feed water, which did not leave the system either from the permeate side or the concentrate side. However, the entire accumulated concentration of cations and anions cannot be assumed as a part of cake-enhanced fouling layer since it also covers the polarization layer. Hence, the mass fraction (%) of cations and anions presented in Figure 4 is conceivably not only the deposited foulants but also the significant stake of polarization layer.

The minimum accumulation of ionic mass is exhibited by Scheme 4, which was operated with CO_2_. It shows the greater efficiency of a membrane in the discharge of cations and anions from the system. The nominal accumulation of ions in Scheme 1 is supposed to be a result of the polarization layer since morphological examination (Section 3.3) did not identify excessive fouling onto the membrane surface. Comparatively, a higher degree of ionic accumulation is observed in other schemes, particularly, in Schemes 2 and 3. This higher accumulation of cations and anions inside the system adversely affects the filtration efficiency of membrane. It is notable that more than 30 and 20% accumulation of divalent cations occurred in Schemes 2 and 3, respectively. The higher degree of Ca^2+^ and Mg^2+^ accumulation in the RO schemes operated with CA-1, CA-2, and CA-3 presented the low efficiency of antifouling agents. Another study also reported the incompetence of commercial antiscalants (combination of phosphonates and carboxylic acids) in controlling the scale formation in RO processes [30]. The fact that the accumulation of foulants on to the surface of RO membrane progressively increases the boundary layer and builds an additional resistance is established. Consequently, the overall membrane resistance increases and the permeate flux decreases [31].

### 3.3. Membrane Surface Analysis

On termination of all of the operational schemes, the surface of all membranes were examined for potential deposition of the foulants. The surface properties and the elemental composition of the membrane were examined by SEM, EDS, and XRD. None of the membrane was found with any structural damage or leakage. However, a viscid deposit was found in the feed spacer channels of the membrane used in Scheme 3. This viscid layer seems to be a part of biofouling, as highlighted by an earlier study [32,33]. It has been noticed in earlier studies that the nutritive role of polyacrylate- and polyphosphonate-derived antifouling agents may cause biofouling during operation of RO membranes [34]. The spacer’s condition contributes significantly to maintaining the consistent permeate flux by RO membrane, and any blockage or malfunctioning in spacers causes serious deterioration in membrane performance [35]. The low performance of operational Scheme 3 can also be related to the membrane spacer issue.

**Figure 5:**
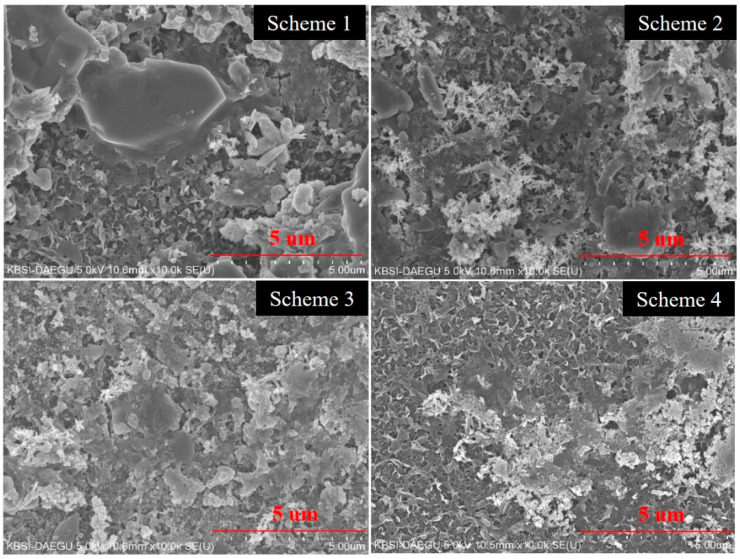
SEM of the fouling deposits on the surface of membranes used in Schemes 1–4.

Figure 5 confirmed the deposition on the surface of membranes. The well-developed calcite crystals were observed in the case of operational Scheme 1. The fibrous structures represented the accumulation of calcium carbonate crystals in combination with other inorganic substances [28]. Both aragonite bunches (with outwardly directed spikes) and calcite crystals (rhombohedral structure) were observed on the surface of membranes. These appearances were found in consistence with reported structures of aragonite and calcite [36]. The slurry-like blotches show the presence of potassium and sodium salts [37]. The in-depth morphological inspection of the deposited crystals confirmed the growth of distinctive plate-like and needle-like gypsum crystals with well-ordered shape and dense structure [38]. The flower-like morphology in Scheme 1 and 2 supposedly appeared because of the accumulated impact of lateral scaling and the bulk deposition on a membrane surface. It has been established that the rate of scale deposition and the morphology of foulants potentially contribute to the loss in permeate flux and fouling resistance [39].

Beside an extensive distribution of foulants onto the membrane surface, the polymeric structure of RO membrane can be easily detected in all the schemes. A prominent difference in surface coverage is observed between Scheme 4 and other schemes. The major surface area of the Scheme 4 membrane was either free of scale or partially covered with scale deposits, which confirms the effectiveness of CO_2_ utilization for scale inhibition in the membrane-based water treatment system. The concentration of multivalent ions in the feed also affects the shape and geometry of crystals.

A study on antiscalants composed of phosphonic acid derivative and polycarboxylic acid confirmed the adsorption of antifouling agent on the nucleation sites of membranes, resulting in variable crystal morphology [28]. A similar study reported the interactions between an acrylate functional group (-COO-) and the Ca^2+^. If Ca^2+^ stays in the neighborhood of acrylate ion, the chances of interacting with SO_4_^2^^−^ is minimal. However, once SO_4_^2^^−^ and Ca^2+^ become close and start nucleation, the acrylate ion of CA-1 and CA-2 remains ineffective at stopping the scale growth [40]. Scheme 3 utilized CA-3, which was composed of polyacrylic acid and PBTC. A study reported the threshold limit of PBTC-derived antifouling agents; however, such antifouling agents did not affect the crystal morphologies [41], as happened in the cases of CA-1 and CA-2. Based on the morphological examination, it can be concluded that the Scheme 4 remained effective at maintaining the saturation of soluble salts at low pH thereby, controlling the scale growth.

**Figure 6:**
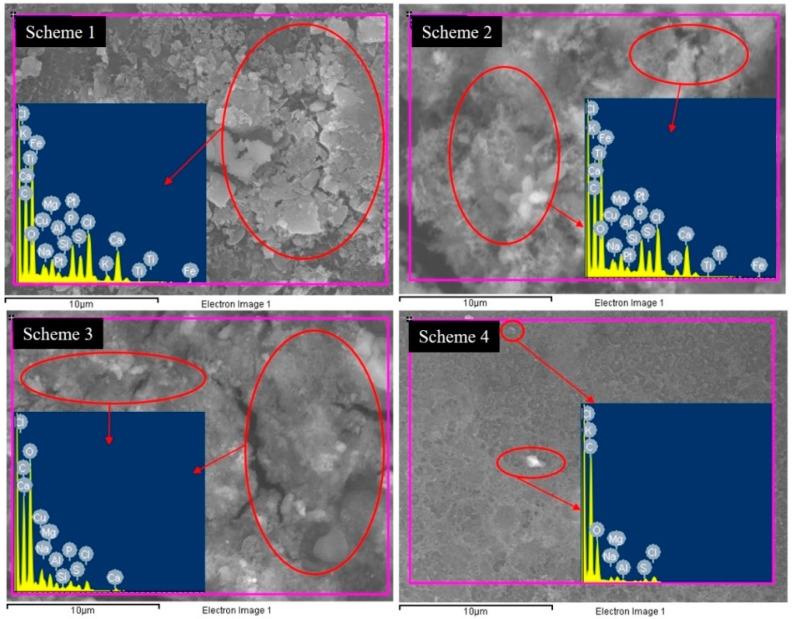
The elemental composition of the scale deposited on the membrane surfaces.

**Figure 7:**
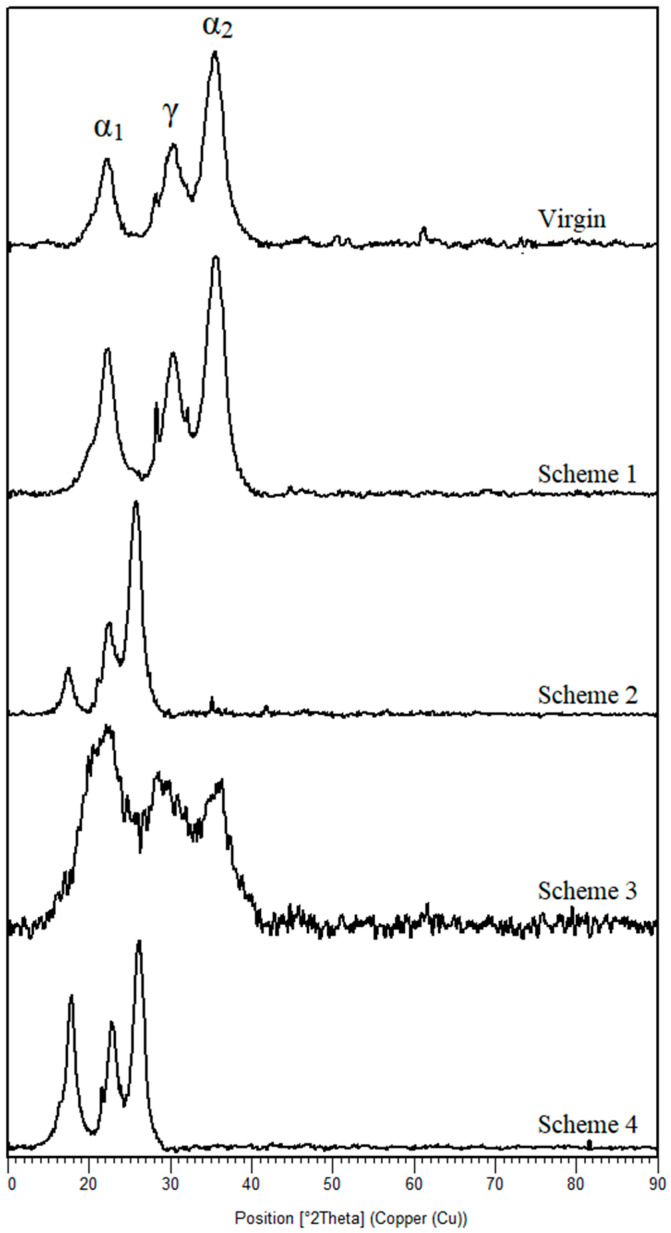
The XRD spectra of virgin and fouled membranes.

The fouling layers identified in SEM analysis were also further confirmed by elemental analysis through EDS. Excluding the basic polymeric elements of polyamide membrane (e.g., oxygen, carbon, and sulfur), several other elements were also identified in EDS spectra of membranes (Figure 6). These elements include calcium, magnesium, sodium, phosphorous, potassium, etc., which are the main constituents of different inorganic salts. The major share of deposition is found in the case of Schemes 1 and 2. Comparatively less deposition is confirmed in the case of Scheme 3. The membranes obtained from Scheme 4 showed a very low rate of deposition, as highlighted in Figure 6. This confirms the effective utilization of CO_2_ for scale inhibition in membrane-based water treatment systems. The comparison of XRD spectra of RO membranes also confirmed the deterioration in basic spectra of virgin membrane after several filtration cycles (Figure 7). The XRD spectra of virgin membrane is found to be consist with the reported XRD pattern of polyamide thin film composite membranes [42]. The intense peaks at 2θ angle of 17.65°, 22.10°, 25.98°, and 41.92° indicated that the crystallinity of polyamide membrane is consist with that in reported studies [43,44]. Both stable crystal forms of polyamide were identified in the virgin membrane, i.e., monoclinic α type and monoclinic (or pseudo-hexagonal) γ type [45]. The intense peaks at 17.65° and 25.98° showed α crystalline phases whereas, the γ crystalline phase is represented by a diffraction peak at a 2θ angle of 22.10°. A most prominent change in the XRD pattern of the Scheme 3 membrane is noticed and confirms the incompetency of PBTC-based antiscalants in inhibiting scale growth. Scheme 4, which operated with CO_2_, did not present any change in the standard XRD pattern of a virgin membrane, which confirms the CO_2_ efficiency in the fouling control.

### 3.4. Cost Assessment

**Figure 8:**
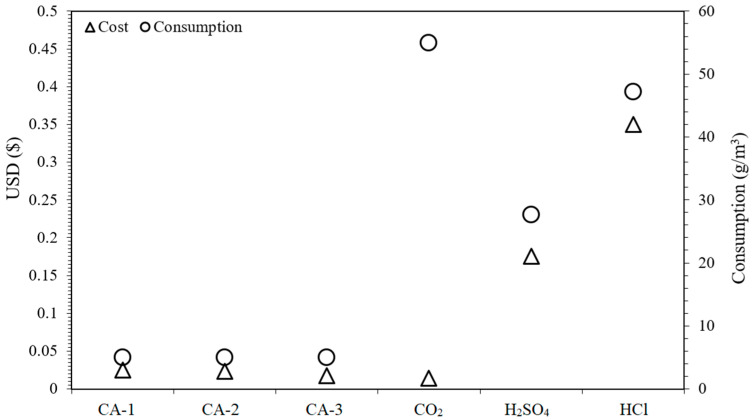
The comparison of consumption cost of antifouling agents during filtration of 1 m^3^ influent.

Based on the operation of different RO schemes, a cost comparison is drawn for different antifouling agents (Figure 8). This cost is calculated considering the added quantity of different antifouling agents during filtration of 1 m^3^ feed water. We also compared the current cost of acid dosage, based on our previously reported study [24]. It is noteworthy that this cost calculation does not involve other operational expenditures such as energy utilization, design, distribution system, pipe lines, labor, overheads, etc. The cost assessment indicated that acidification involves the highest cost compared with CO_2_ and the commercial scale antifouling agent. Furthermore, the cost of CO_2_ also remains low compared with antiscalants when it is utilized for lowering the pH of an influent from ~7 to ~6. It is also notable that the optimal CO_2_ utilization is highly important for a cost effective operation. The higher dosing rate of CO_2_ may increase the cost to even higher than the antiscalants. It is noteworthy as an excessive amount of CO_2_ is required for lowering the pH below 6, which is not suitable in terms of cost effectiveness [46].

CO_2_ utilization can also minimize other operational costs including membrane lifetime and replacement cost, CIP cost (frequency, cleaning agents, and their volume), concentrate disposal cost, energy needs, etc. The membrane replacement accounts for 25–40% of the entire cost in a membrane plant [47]. The life of membrane depends on the extent of membrane aging and destruction. As a green antiscalant, CO_2_ minimizes the fouling in membrane plants and, hence, improves the permeate flowrate, reduces the irreversible membrane damage, and decreases the operational costs of frequent CIP. The permeate flux is the most influential parameter as it is directly associated with productivity and determines the pressure (consequently the energy needs). The application of antiscalants may produce some byproducts in the RO concentrate, which poses severe risk to the environment and the ecosystem, leading to a higher concentrate disposal cost. This environmental hazard and associated impact on cost can be neutralized by CO_2_ utilization in membrane-based water treatment systems.

## 4. Conclusions

This study compares CO_2_ utilization to improve the efficiency of membrane-based water treatment systems by reducing the fouling potential. The primary reason for CO_2_ utilization is to take advantage of lowering the pH of RO and to kinetically control the precipitation of inorganic salts onto the surface of membrane. The performance of different operational schemes was compared in the presence of CO_2_ and other commercial antifouling agents such as CA-1, CA-2, and CA-3. The percent salt rejection and permeate flux profile of all the schemes were compared. The efficiency trend for all the schemes can be depicted as Scheme 4 > Scheme 3 > Scheme 1 > Scheme 2. The percent salt rejection and flux profile confirmed the superiority of CO_2_ utilization on other antiscalants in terms of scale inhibition. This efficiency is further confirmed by the membrane surface analysis. The SEM, EDS, and XRD results confirmed the suitability of membrane structure and minimum scale deposition in Scheme 4 compared with other schemes. In conclusion, CO_2_ is presented as a green antifouling agent for membrane-based water treatment systems. Several antiscalants may generate toxic byproducts in RO concentrate, which pose a serious risk to the environment and the ecosystem. This threat can be evaded with the application of CO_2_ as a green antiscalant. Moreover, the greenhouse gas reduction effects can be expected via CO_2_ utilization in water treatment systems. The cost assessment showed that the utilization of CO_2_ is quite economic compared with antiscalants and acidic solutions for scale inhibition in RO operation. Hence, the cost effective and environmental friendly CO_2_ utilization approach can significantly contribute in enhancing the sustainable circular economy.

## Figures and Tables

**Table 1:** Quality of synthetic wastewater used in this study.

Parameter	Analytical Result	Parameter	Analytical Result
pH	7.11–7.25	Conductivity	1850–2450 µS/cm
Na^+^	470–490 mg/L	K^+^	50 ± 5 mg/L
Ca^2+^	190–210 mg/L	Mg^2+^	85–105 mg/L

## Data Availability

The data presented in this study are available in the article.
